# Bulimia symptoms in Czech youth: prevalence and association with internalizing problems

**DOI:** 10.1007/s40519-019-00790-w

**Published:** 2019-10-16

**Authors:** Anna Larsen, Marie Lilja, Knut Sturidsson, Marek Blatny, Michal Hrdlicka, Andrew Stickley, Vladislav Ruchkin

**Affiliations:** 1 Division of Psychology, Department of Clinical Neuroscience, Karolinska Institute, Stockholm, Sweden; 2 Department of Psychology, Faculty of Arts MU, Brno, Czech Republic; 3 Department of Child Psychiatry, Charles University Second Faculty of Medicine, University Hospital Motol, Prague, Czech Republic; 4 Stockholm Center for Health and Social Change (SCOHOST), Södertörn University, Huddinge, Sweden; 5 Child and Adolescent Psychiatry Unit, Department of Neuroscience, Uppsala University, 751 85 Uppsala, Sweden; 6 Child Study Center, Yale University Medical School, New Haven, CT 06520 USA; 7 Säter Forensic Psychiatric Clinic, 783 27 Säter, Sweden

**Keywords:** Bulimia symptoms, Internalizing problems, Prevalence, Czech Republic, Adolescents

## Abstract

**Objective:**

Although clinical studies suggest that bulimia symptoms are common in youth, research on the prevalence of such symptoms and of their association with comorbid internalizing problems in the general population has been limited. This study aimed to evaluate the gender-specific prevalence of bulimia symptoms in Czech youth and explored the association between a clinical level of self-reported bulimia symptoms (CLBS) and internalizing problems by gender, controlling for age, socio-economic status and puberty status.

**Method:**

The study was conducted on a representative national sample of Czech youth (*N *= 4430, 57.0% female) using self-report scales. Multivariate analysis of covariance (MANCOVA) was used to examine the associations.

**Results:**

The 3-month CLBS prevalence was higher in girls (11.4%) than in boys (3.8%) and in both genders a CLBS was associated with higher levels of comorbid internalizing problems.

**Discussion:**

Timely recognition of bulimia symptoms and associated risk factors is important for early prevention and intervention strategies.

**Level of evidence:**

V, cross-sectional descriptive study (according to Oxford (UK) CEBM Levels of Evidence, 2011).

## Introduction

In recent years, there has been a plethora of clinical studies addressing a broad range of factors relating to eating disorders (ED). It is well documented that ED are associated with a number of adverse physical, social and psychological consequences [1]. They are difficult to treat, impose a significant burden on health services and have one of the highest mortality rates of all psychological disorders [2]. Bulimia, defined by an overvaluation of weight, shape and the behavioral symptoms of recurrent binge eating accompanied by purging and fasting, is one of the most common ED [3], although until now, the prevalence of bulimia symptoms in the general population, especially outside the US, has been less well investigated [4] (see, however, [5–8]).

While a recent review of the literature on ED in children and adolescents [9] suggests a lifetime prevalence of anorexia between .5 and 2%, of bulimia between .9 and 3%, and of eating disorders not otherwise specified (EDNOS, a group of heterogeneous disorders consisting primarily of subclinical anorexia and bulimia symptoms) of 4.8%, those community studies that have utilized dimensional measures have produced even higher prevalence estimates of disordered eating behaviors, between 14 and 22% [10, 11]. Similar to other eating problems, bulimia symptoms tend to be chronic [3, 12] and are associated with a high rate of hospitalization, outpatient care, and emergency department visits, resulting in a high cost to society [13]. Although, the point prevalence of the disorder peaks in older adolescence or young adulthood [14], bulimia symptoms are becoming more common in younger age groups [3, 15], although as yet, research examining prevalence rates in adolescents from the general population has been limited [e.g. 3, 5, 16–18].

As with other ED, adolescent girls are most at risk of developing bulimia symptoms [7, 19], with the female-to-male ratio estimates varying between 3:1 [18] and 10:1 [20, 21]. However, there is a lack of population-based data on weight concerns and disordered eating behaviors among adolescent males [22, 23].

Longitudinal studies have found that weight and shape concerns and weight control behaviors are potent predictors of the onset of ED [24–26], while other research has suggested that weight and shape concerns predict the degree of psychosocial impairment in bulimia [27]. There is also some evidence that the onset of bulimia symptoms may be linked to higher body dissatisfaction and a greater body-mass index (BMI) [26, 28], although the latter doesn’t seem to be related to either the severity of bulimia symptoms [29], or to psychosocial impairment related to such symptoms [27]. In addition, increased levels of disordered eating behaviors (including bulimia symptoms), have been associated with both early pubertal timing and more advanced pubertal development [30].

While historically, there has been a stereotypical belief that ED, including bulimia, mostly affect young, white females with a higher socio-economic status [31], this perspective has been challenged by recent research. In particular, some authors have postulated that the onset of eating disorders in adolescents is not associated with the socio-economic status of their families [32], while others have suggested that a lack of food and economic hardship may be inversely associated with the development of eating problems [33].

Previous research has described high levels of comorbid problems associated with ED in general [18] and suggested a close link between bulimia symptoms and internalizing problems in particular [34], especially with regard to anxiety and depressive symptoms [35–37], posttraumatic stress disorder (PTSD) [18], as well as substance abuse [6, 38]. Indeed, it has been suggested that the prevalence of comorbid mood disorders may be higher in bulimia patients than in anorexia patients [39], while some studies have indicated that early depressive symptoms may predict the development of bulimia symptoms [35, 40]. In addition, all eating disorders are associated with a significantly higher likelihood of self-harm, suicide, and death [41]. Other recent studies have emphasized a significant overlap in symptom patterns and associated personality traits between ED, including bulimia, obsessive–compulsive disorder and autism spectrum disorders [33, 42], while other research has shown that bulimic-spectrum disorder has significantly elevated levels of impulsivity when compared with restrictive disorders [41].

While providing an important insight on the prevalence of bulimia symptoms, as well as on their association with mental health problems, previous studies have a number of shortcomings, which limit their generalizability. Investigations of bulimia symptoms, as well as other ED in youth community samples have been relatively rare, with studies tending to exclude those youth who fall below current diagnostic thresholds [18], even though this group tends to have higher rates of comorbid psychopathology and has an increased risk for clinically significant disordered eating [36]. In addition, although disordered eating behaviors tend to develop relatively early, with some studies suggesting that the median age of onset among adolescents is 12- to 13-years [18], most of the previous population-based prevalence studies have focused on young adults (18+) [3] or mid-adolescents (14–16 years) [43, 44]. Furthermore, many studies have been centered primarily on females, while disordered eating behaviors in males have been generally described as “underdiagnosed, undertreated and misunderstood” [45].

Hence, the aims of the present study were: (1) to examine the gender-specific prevalence of bulimia symptoms in a nationally representative sample of adolescents from the general population and to explore their association with puberty status and BMI, and (2) to determine whether the association between a clinical level of bulimia symptoms (CLBS) and internalizing problems is gender specific, while controlling for age, socio-economic status (SES) and puberty status. The reason for using a CLBS for the analyses was to be able to set a meaningful cut-off i.e. where although many of those who were classified as cases would not have been clinically diagnosed with the disorder, they would nevertheless still have a self-reported symptom level corresponding to it. Gaining a better understanding of individuals who may have the disorder, but do not seek help, is an important issue, especially considering that many of them may have other psychiatric problems.

## Method

### Participants

The data used in this study came from a school survey undertaken in the Czech Republic. A total of 4703 reports were collected, but 273 students were excluded from the original sample because of missing values on any variables of interest (*N *= 198), as well as because some were older than 17 years of age (*N *= 75). The youths in the excluded group were slightly older than those included in the analyses, *M* (*SD*s) = 15.45 (2.46) vs. 14.63 (1.67) years, *t* = 2.86, *p *< .01. In addition, the excluded group reported higher levels of posttraumatic stress [*M* (*SD*s) = 25.37 (11.94) vs. 22.67 (11.00), *t* = 3.92, *p *< .001], and slightly higher levels of depressive symptoms [*M* (*SD*s) = 4.92 (3.88) vs. 4.41 (3.94), *t* = 1.99, *p *< .05]. Otherwise, the groups did not differ from each other on any other variables of interest. The final sample consisted of 4430 students.

Participants in the study sample ranged in age from 12 to 17 years [*M* (*SD*) = 14.63 (1.66)]. The composition of the sample was 57.0% female (*N *= 2523), an accurate reflection of the local public school population. Boys, as compared to girls, had a significantly higher BMI [20.08 (3.06) vs. 19.58 (2.82), *t* = 5.07, *p* < .001], but did not differ in terms of age [14.59 (1.65) vs. 14.66 (1.68), *t* = 1.32, ns). Most of the participants (79.6%) came from two-parent families. According to the students’ reports, 58.2% of their fathers and 41.8% of mothers had completed the equivalent of a high school education or beyond.

Ethical approval for the study was obtained from the ethical committee at the Academy of Sciences of the Czech Republic. It was performed in accordance with the ethical standards laid down in the 1964 Declaration of Helsinki and its later amendments.

### Procedure

Stratified probability sampling of schools, according to location and school type, was conducted to identify a national probability sample of youth in the Czech Republic. Only general public schools were included in the study (i.e. schools with special education programs were left out). The students that participated belonged to classes that had been randomly selected within randomly selected schools (*N *= 150). Students completed the survey in their classrooms during a regular school day. The questionnaire was administrated in paper and pencil form by trained administrators with written informed consent being obtained from each participant. Prior to the study administration, the parents of the students were informed of the study by mail, as well as of the possibility to decline the participation of their child. Children also had the right to refuse participation at the time of the survey’s administration with those children who refused being provided with alternative tasks. A total of 1.4% of the students or their parents refused participation.

### Measures

*Disordered eating behaviors* were assessed using a shortened version of the Eating Disorder Diagnostic Scale originally developed by Stice et al. [46]. The scale measures the occurrence of disordered eating thoughts and behaviors during the previous 3 months. The scale consists of four statements addressing the occurrence of anorexia and bulimia symptoms, namely: “I worried a lot about how to stop gaining weight” (anorexia/bulimia), “I felt fat even when others told me I am too thin” (anorexia/bulimia), “I felt very upset about my overeating or weight gain” (bulimia) and “I ate large amounts of food even when I didn’t feel hungry” (bulimia). Response options were: “Not true” (scored 0), “Somewhat true” (1), and “Certainly true” (2). The internal consistency of this scale was acceptable (Cronbach’s *α* = .74). To assess the frequency of these thoughts and behaviors, there were two questions enquiring about how many times per week the respondent engaged in certain behaviors with the purpose to prevent weight gain, including (1) vomiting or the use of laxatives and (2) fasting (skipping at least 2 meals in a row) or engaging in excessive exercise. These were answered on a five-point scale ranging from “0 times” (scored 0) to “More than 10 times” (scored 4).

We used positive responses (Somewhat true or Certainly true) on the items above for coding while using DSM-5 criteria for bulimia [47], so that a *proxy for a possible bulimia diagnosis* i.e. a CLBS variable was created. The diagnostic criterion A (Recurrent episodes of binge eating) was coded based on the item: I ate large amounts of food, even when I didn’t feel hungry. The B and C criteria (Recurrent inappropriate compensatory behaviors, such as vomiting, use of laxatives, fasting or excessive exercise, that occur to prevent weight gain at least once a week for 3 months) were assessed using two items: About how many times per week have you made yourself vomit or used laxatives to prevent weight gain? (at least once) OR About how many times per week have you fasted (skipped at least two meals in a row) or engaged in excessive exercise to prevent weight gain? (at least once). Criterion D (Self-evaluation is unduly influenced by body shape and weight) was coded using a positive response for either of the following statements: I felt fat even when others told me I am too thin, OR I felt very upset about my overeating or weight gain. Finally, criterion E (the disturbance does not occur exclusively during an episode of anorexia nervosa) was applied (including use of the age-relevant BMI cut-offs for underweight). Using positive symptom scores for all five diagnostic criteria, a binary variable was created (0/1), as a proxy for bulimia. This variable was used in all further analyses and was denoted as the Clinical Level of Bulimia Symptoms (CLBS).

*Depressive symptoms* were assessed using an adaptation of the *Center for Epidemiologic Studies*-*Depression Scale* (CES-D). Both the CES-D [48] and several modified versions of it [e.g. 49, 50] have demonstrated excellent psychometric properties with adolescent populations. The scale consists of ten negative statements (e.g. “I felt I could not shake off my sad feelings even with help from my family or friends”; “I felt really down”). Respondents reported on the presence of depressive symptoms during the past month using a three-point scale [“Not true” (scored 0); “Somewhat true” (1); or “Certainly true” (2)]. The total score could range from 0 to 20, with higher scores indicating higher levels of depressive symptoms. The scale had good internal consistency (Cronbach’s *α* = .83).

*Anxiety symptoms* were assessed with a 12-item scale [51] which included questions characterizing worrisome or preoccupying thoughts and feelings (e.g. “I worry about how well I do things”, “I avoid going to unfamiliar places”), with a three-point answer scale [“Not true” (scored 0); “Somewhat true” (1); or “Certainly true” (2)]. The total score could range from 0 to 24, with higher scores indicating more anxiety symptoms. The scale had good internal consistency (Cronbach’s *α* = .87).

*Posttraumatic stress* was assessed by the Child Post-Traumatic Stress—Reaction Index (CPTS-RI). This self-report questionnaire [52] contains 20 items with a 5-grade response scale: “Never” (scored 0),” A little” (1), “Sometimes” (2), “Often” (3), or “Most of the time” (4). A total score between 12 and 24 indicates mild Post-Traumatic Stress Symptoms (PTSS), a score of 25–39 indicates moderate PTSS, 40–59 severe PTSS, and a score of 60 or above indicates very severe PTSS. CPTS-RI scores have been found to correspond closely with an actual clinical diagnosis of posttraumatic stress disorder (PTSD) based on an interview [53]. Cronbach’s alpha for the scale was .81.

*Somatic complaints* were assessed using a 10 item-scale that examines somatic complaints commonly reported by children and adolescents [54]. The respondents reported on the presence of somatic symptoms during the past month on a three-point scale [“Not true” (scored 0); “Somewhat true” (1); or “Certainly true” (2)]. The total score could range from 0 to 20, with higher scores indicating increased somatic symptoms. The internal consistency of the scale was good (Cronbach’s *α* = .83).

*Puberty scale* items were adapted from a pubertal development measure [55]. The items asked whether participants experienced “oily skin”, “pimples”, “underarm hair”, “voice change”, “changing color or amount of leg hair” and “fast body growth”. Positive responses were coded as 1 and negative responses as 0. Responses to each of the 6 items were summed to create a continuous scale running from 0 to 6 with higher scores indicating more advanced pubertal development.

*Proxy for low  socioeconomic status (SES)* Family structure was re-coded for the analyses into a binary variable based on student reports of coming from a single-parent family or otherwise (1 vs. 0). Parental education was recoded into a binary variable (1 = lower than college education) and was calculated separately for each parent. Finally, parental employment status was also assessed for each parent separately (2 = unemployed, 1 = part-time employed, 0 = full-time employed). A proxy for SES was calculated as the sum of the above variables (single-parent family status, parental education and parental employment status), hence producing a variable potentially ranging from 0 to 6, with higher scores indicating lower SES.

*Body-mass index (BMI)* Using student-reported height and weight we calculated a BMI score for each student.

### Statistical analyses

Data were analysed using the Statistical Package for the Social Sciences (SPSS-25.0). Chi-square and independent sample *t* tests were used for univariate comparisons of demographic characteristics and of dependent variables by gender. General linear models (GLM) multivariate analysis of covariance (MANCOVA) was then used to determine main and interaction effects across the fixed factor of a CLBS (1/0) (as described earlier) and gender (boys = 1, girls = 0), while adjusting for the covariates of age, puberty, and SES.

Two separate MANCOVA analyses were conducted for the internalizing group of variables (depression and anxiety symptoms, somatic complaints, and posttraumatic stress), henceforth referred to as internalizing problems. Thus, we used a 2 (CLBS) × 2 (gender) design for assessing differences in internalizing problems. The unique contribution of each of the two fixed factors, the four covariates, and the one interaction term were assessed through follow-up between-subject tests and unstandardized parameter estimates derived from the MANCOVA. Results are presented as means (*M*) and standard deviations (*SD*), and for individual outcomes, as partial eta squared (*η*^2^), a common metric of effect size that represents the unique amount of variance explained by each predictor variable.

## Results

A large number of adolescents of both genders reported experiencing disordered eating behaviors, including bulimia symptoms, in the past 3 months. Table 1 presents the descriptive statistics [*M* (*SD*)] for the Chi-square tests comparing the prevalence of specific disordered eating behaviors by gender. The symptom prevalence varied substantially from 1. 2% [for making oneself vomit or using laxatives to prevent weight gain (in boys)], to 48% [for feeling very upset about overeating or weight gain and for feeling fat even when others told the opposite (both in girls)], to 62.8% [for worrying a lot about how to stop gaining weight (in girls)]. The prevalence of all disordered eating behavior symptoms was significantly higher in girls than boys. When comparing other variables of interest (see Table 3 for M(SD)), boys (as compared to girls) reported lower levels of depressive symptoms, *t *= 15.58, *p* < .001; of anxiety, *t *= 14.25, *p* < .001; of somatic complaints, *t *= 14.34, *p* < .001, of posttraumatic stress, *t *= 18.06, *p* < .001, and of disordered eating behaviors *M* (*SD*s) = 1.23 (1.62) vs. 2.74 (2.26), *t *= 25.09, *p* < .001 (data for the latter  not shown in the table). The prevalence of a CLBS was also significantly higher in girls [287 (11.4%)] than in boys [73 (3.8%)] Chi square = 82.95, *p *< .001). Both boys and girls with a CLBS generally reported a more advanced puberty status overall and higher BMI levels than those without a CLBS (Table 2).

**Table 1:** Prevalence of different types of disordered eating behaviors in the past 3 months by gender (*N* (%))

During the past 3 months	Boys	Girls	*χ*^2^, *p*
I worried a lot about how to stop gaining weight	444 (23.3)	1585 (62.8)	684.00; < .001
I felt fat even when others told me I am too thin	311 (16.3)	1219 (48.4)	492.57; < .001
I ate large amounts of food even when I didn’t feel hungry	747 (39.3)	900 (35.8)	5.62; < .05
I felt very upset about my overeating or weight gain	313 (16.4)	1229 (48.8)	500.22; < .001
I made myself vomit or used laxatives to prevent weight gain (*at least once per week*)	22 (1.2)	103 (4.1)	34.06; < .001
I fasted (skipped at least 2 meals in a row) or engaged in excessive exercise to prevent weight gain (*at least once per week*)	258 (13.5)	798 (31.6)	195.98; < .001

Prevalence described for “somewhat true” or “certainly true” responses, unless indicated otherwise

**Table 2:** Results of one-way ANOVA tests [M(SD)] comparing puberty and BMI by gender and CLBS

	CLBS		No CLBS		Statistics
	Boys (1)	Girls (2)	Boys (3)	Girls (4)	
Puberty^a,b,c,d^	3.93 (1.64)	3.13 (1.37)	3.67 (1.65)	2.93 (1.40)	*F* = 87.67; *p* < .001
BMI^b,c,d,e,f^	21.39 (3.71)	20.91 (2.87)	20.03 (3.02)	19.41 (2.77)	*F* = 38.90; *p* < .001

Letters in superscript denote significant differences: a—between 1 and 2; b—between 3 and 4; c—between 1 and 4; d—between 2 and 3, e—between 1 and 3; f—between 2 and 4

*CLBS* clinical level of bulimia symptoms

### CLBS and internalizing problems

When assessing the differences in internalizing problems by a CLBS (see Table 3 for descriptive statistics [*M* (*SD*)] by gender and Table 4 for the tests of between-subjects effects) the main effect for the model was significant (Wilks’ lambda = .925; *F* (4, 4430) = 89.31, *p *< .000, *η*^2^ = .075). As concerns specific effects, the main effect for a CLBS was significant (Wilks’ lambda = .960; *F* (4, 4430) = 46.49, *p *< .000, *η*^2^ = .040), with increased levels of internalizing problems in those with a CLBS. The main effect for Gender was significant (Wilks’ lambda = .985; *F* (4, 4430) = 16.55, *p *< .000, *η*^2^ = .015), demonstrating higher levels of internalizing problems for girls (see Table 4).

**Table 3:** Internalizing problems [M (SD)] by bulimia symptoms in boys (B) and girls (G)

	CLBS		Total group
	Yes	No	
Depressive symptoms			
B	6.10 (4.69)	3.29 (3.20)	3.40 (3.31)
G	7.69 (4.64)	4.91 (4.07)	5.23 (4.23)
Anxiety			
B	11.76 (5.58)	8.58 (4.66)	8.70 (4.74)
G	12.47 (4.80)	10.48 (4.61)	10.71 (4.68)
Somatic complaints			
B	6.91 (4.56)	3.87 (3.28)	3.99 (3.39)
G	7.47 (4.06)	5.30 (3.64)	5.55 (3.75)
Posttraumatic stress			
B	27.40 (13.56)	19.08 (9.36)	19.39 (9.68)
G	32.14 (11.65)	24.30 (10.96)	25.19 (11.32)

*CLBS* clinical level of bulimia symptoms

**Table 4:** Effect sizes for each dependent variable (internalizing problems) (*η*^2^, *p*)

	Depressive symptoms	Anxiety symptoms	Somatic complaints	Posttraumatic stress
Age	.004, < .001	.001, < .05	.001, ns	.003, < .01
Puberty	.010, < .001	.009, < .001	.012, < .001	.007, < .001
SES	.000, ns	.001, < .05	.001, ns	.005, < .001
Gender	.010, < .001	.005, < .001	.005, < .001	.012, < .001
CLBS	.026, < .001	.014, < .001	.025, < .001	.028, < .001
CLBS by gender	.000, ns	.001, ns	.001, ns	.000, ns

*CLBS* clinical level of bulimia symptoms

The main effect for Age was also significant (Wilks’ lambda = .990; *F* (4, 4430) = 10.62, *p *< .000, *η*^2^ = .010), as was the main effect for Puberty (Wilks’ lambda = .983; *F* (4, 4430) = 19.43, *p *< .000, *η*^2^ = .017), suggesting increasing levels of internalizing symptoms along with increasing age and pubertal development. The main effect for SES was also significant (Wilks’ lambda = .994; *F* (4, 4430) = 7.11, *p *< .001, *η*^2^ = .005), indicating differences in internalizing problems in relation to socio-economic status. With regard to the interaction effects, the interaction effect for CLBS × gender was not significant (Wilks’ lambda = .999; *F* (4, 4430) = 1.65, n.s., *η*^2^ = .001), which means that the patterns of internalizing problems in relation to a CLBS were not gender-specific. Table 4 presents effect sizes for each dependent variable (depression and anxiety symptoms, somatic complaints, and posttraumatic stress). In both genders, anxiety and depression symptoms, somatic complaints and posttraumatic stress similarly increased in relation to a CLBS.

As the differences by outcome might have been masked by use of the MANCOVA analysis (i.e. by simultaneously assessing several outcomes in one model), each outcome was examined separately to determine whether the results that were obtained from the MANCOVA were the same for each individual outcome. This produced very similar results.

## Discussion

This study showed that a considerable number of adolescents reported bulimia symptoms during the past 3 months and that the prevalence of bulimia symptoms differed significantly by gender. Our results suggest that individual ED symptoms are common in youth, especially in girls, and that the worries associated with potential weight gain and a distorted body image are highly prevalent. At the same time, the number of adolescents that actually attempted to correct a perceived weight gain or distorted body image with compensatory strategies (vomiting, use of laxatives) was more limited. This finding can be potentially explained by the general worries concerning body image typical for adolescents [56]. In addition, those with a CLBS had a significantly higher BMI when compared to those without a CLBS (although both were within age norms), which could have caused them greater concerns about their own weight, as adolescents commonly tend to compare themselves to their peers [56]. As weight and shape concerns, higher body dissatisfaction and weight control behaviors tend to predict the onset of ED [24–26, 28] then gaining a better awareness of the prevalence of such concerns is important. The finding that the CLBS prevalence was higher among girls than boys accords with earlier research, which has shown that bulimia symptoms are more frequent in adolescent girls [18, 20, 21].

In line with previous studies we also found that the presence of disordered eating behavior in youth in the general population tends to be associated with greater BMI. Although this association was found in both boys and girls, and boys in general had a somewhat greater BMI, girls generally expressed more weight concern than boys in relation to BMI. This might have several explanations. While males are more likely to have a history of higher weight prior to ED onset [57], research shows that men tend to be more satisfied with their bodies than women [58] and that the ideals for men are about muscularity and leanness [59, 60], rather than thinness, which is the ideal for women. The fact that women are largely subjected to an idealization of thinness [61], expectations from society concerning an unrealistic appearance [62] and pressure to be thin from different forms of media [63, 64] could contribute to body dissatisfaction when at a healthy BMI range. Furthermore, previous research indicates that women are more likely than men to judge themselves as being overweight even when they are not, suggesting that judgements about weight are affected by unhealthy standards [56].

The combined prevalence of bulimia symptoms that could potentially constitute a disorder (CLBS—3.8% in boys versus 11.4% in girls) was substantially lower than the prevalence of the individual bulimia symptoms, but still higher than the prevalence of a clinical diagnosis of the disorder in the general population reported in previous studies, where estimated prevalence rates have ranged between .9% and 3% for bulimia, and were 4.8% for EDNOS [8]. It is important to keep in mind that the presence of a CLBS was evaluated based solely on a self-report scale (that included all affirmative answers, i.e. both somewhat true and certainly true responses), which might help explain why the CLBS prevalence in this study was similar to the previously reported rates of subclinical disordered eating, which is usually far more common [17, 65, 66]. At the same time, the fact that there were so many adolescents who had a CLBS and who correspondingly used compensatory strategies for decreasing weight is alarming. This highlights why understanding the patterns of subclinical disordered eating behavior is of clinical importance especially as it has been linked to poor health-related quality of life, as well as higher rates of comorbid psychopathology, and an increased risk for clinically significant disordered eating [36].

The substantial prevalence of subclinical symptoms may potentially suggest a large degree of symptom fluctuation in adolescence, where symptom counts do not necessarily reach the level of a disorder, but still increase the risk for it and for comorbidity. This may also indirectly support the notion of a potential dimensional continuum between different kinds of ED, as well as other possible pathways that might underlie both bulimia and internalizing behaviors e.g. genetic factors possibly producing different phenotypes of ED depending on environmental factors [33, 67].

The finding that a CLBS was correlated with internalizing problems accords with findings from previous research (e.g. 34). Individuals with eating disorders often suffer from primary anxiety and depressive disorders that began before the onset of the ED [35, 37, 68] and demonstrate high levels of comorbid anxiety and mood disorders [18]. A history of trauma has been documented in many patients with ED [e.g. 69] and traumatic experiences have long been considered a significant, albeit non-specific, risk factor for disordered eating behaviors [68]. Two major studies with adults have reported significantly higher rates of PTSD in individuals with bulimia nervosa [3, 70], while Swanson et al. [18] found that adolescents with bulimia nervosa were 7.6 times more likely to have comorbid PTSD. In addition, there is some evidence that patients with disordered eating behaviors may be more vulnerable to stress and its consequences, as well as exhibit high levels of anxiety sensitivity in general [68]. As individuals with an ED complicated by PTSD require treatment for both conditions, using a trauma-informed, integrated approach [71] then not addressing trauma during the treatment of an eating disorder makes it more likely that successful recovery will be thwarted [68].

The results also revealed a gender-specific difference in internalizing problems in general, with boys reporting lower levels of internalizing problems than girls. This is in line with previous research indicating that girls tend to internalize their problems [72, 73]. It should be mentioned, however, that in spite of these gender differences in internalizing problems, the patterns of internalizing problems in relation to a CLBS were not gender-specific, that is, both boys and girls with a CLBS reported similarly increased internalizing problems, as compared to boys and girls without a CLBS. This is in line with previous reports suggesting that males and females report similar levels of overall psychological distress and impaired quality of life associated with disordered eating behaviors [57].

## Limitations

Several limitations should be kept in mind when considering the findings of this study. First, as the data were cross-sectional it was not possible to establish causality or even determine the temporal order of the observed relations. Second, the use of adolescents’ self-reports for the main independent and dependent variables without being able to verify the accuracy of this information means that the data may be subject to different biases, such as reporting bias, recall bias and social desirability bias. Third, the study did not assess whether the students had a previous diagnosis and/or previous or current treatment for bulimia or any other ED and/or other mental health problems. In addition, the study did not assess whether bulimia symptoms were associated with clinical impairment. This is important as it is likely that many of the adolescents, who were categorized as having a CLBS, would not meet the diagnostic criteria for bulimia in clinical terms, making the results less generalizable to clinical populations.

## Conclusions

This cross-sectional study of bulimia symptoms in Czech youth found that a CLBS was common among adolescents, with the 3-month CLBS prevalence rate being 11.4% in girls, and 3.8% in boys, and that internalizing problems were more common among adolescents in the CLBS group, compared to other students. The first step in a process of helping individuals with bulimia symptoms is to recognize their symptoms at an early stage and respond to them in a timely manner. In connection with this, our study increases awareness that bulimia symptoms can be experienced in younger age groups and that hence, the timely identification of those in need of help is essential. Future research could potentially explore whether the association differs between different bulimia symptoms and different internalizing outcomes and more clearly specify these associations in different adolescent populations.
